# Neural processing of emotion in multimodal settings

**DOI:** 10.3389/fnhum.2014.00822

**Published:** 2014-10-21

**Authors:** Martin Klasen, Benjamin Kreifelts, Yu-Han Chen, Janina Seubert, Klaus Mathiak

**Affiliations:** ^1^Department of Psychiatry, Psychotherapy, and Psychosomatics, Medical School, RWTH Aachen UniversityAachen, Germany; ^2^Jülich Aachen Research Alliance-Translational Brain Medicine, RWTH Aachen UniversityAachen, Germany; ^3^Department of Psychiatry and Psychotherapy, University of TuebingenTuebingen, Germany; ^4^Department of Psychiatry, The University of New Mexico School of MedicineAlbuquerque, NM, USA; ^5^Psychology Division, Department of Clinical Neuroscience, Karolinska InstitutetStockholm, Sweden

**Keywords:** emotion, multisensory integration, social environment, EEG, fMRI

Perhaps the most astonishing outcome of the Research Topic *Neural processing of emotion in multimodal settings* was the wide resonance. Not too long ago, emotions as well as multisensory integration both played outsider roles in neuroscience. However, nowadays the processing of emotional signals in the human brain has become an integrative part of basic neuroscience and clinical research. Considered a mere side effect of reasoning and thinking, the importance of emotions for human behavior has been underestimated for many years. The discovery of complex brain systems dedicated to the detection of harmful or positive situations, emotion recognition in others, and emotional experience have led to the conclusion that emotions are not at the periphery, but at the very core of human behavior. Among others, facial expressions, gestures, postures, and prosody express emotions. Thus, their integration is an essential part of face-to-face social interactions (De Gelder and Vroomen, 2000). Therefore, emotions have been described as inherently multimodal (Robins et al., 2009). This is also reflected on the psychological level, e.g., congruent bimodal emotions lead to shorter reaction times compared to faces alone (Massaro and Egan, 1996; Dolan et al., 2001).

Reflecting their evolutionary significance, emotional stimuli undergo preferred processing in the human brain (Klasen et al., 2011, 2012a). Emotion-relevant cues are delivered via multiple modalities: A picture of a beloved person evokes pleasant feelings; the furious barking of a dog signals danger; disgusting smell or taste helps to identify spoiled food. More than this, emotional cues mostly appear in combination: We recognize panic in another person by a fearful face and a frightened voice, but also by less obvious cues such as the perception of fear sweat. However, research has begun only recently to address behavioral and neural aspects of emotion integration (Klasen et al., 2012a). The aim of this volume is to fill in this gap. The studies reported here present a wide range of emotional stimuli—social and non-social—spanning the whole range of sensory modalities, from auditory and visual to touch and chemosensation.

Despite a considerable body of neuroimaging literature on emotion processing, the pathways of emotional information in the human brain are not fully understood. Considering multisensory emotions raises the additional question how these streams are integrated. Freiherr et al. (2013) provide an overview over sensory integration aspects and their development with healthy aging. Recent neurobiological models propose multiple interactions between cortical and subcortical stuctures (Senkowski et al., 2008). Social emotion processing, however, is complex and involves bottom-up processes and top-down modulations. The full understanding of this complex interplay calls for methods that identify areas of emotional integration, but also show the time course and flow of information. Given the spatial proximity of unisensory and multisensory integration areas, there is a need for high resolution data in both time and space. The new technique of simultaneous EEG and fMRI recordings may adequately address this issue. Schelenz et al. (2013) present a novel source-localization driven analysis for EEG-informed fMRI. Applied to multisensory emotion paradigms, this method has the potential to map the exact cortical pathways of audiovisual signal integration.

Social emotion processing is disturbed in some clinical populations. In some psychiatric conditions this may even lie at the core of the affective symptomatology. Accordingly, a multitude of studies have addressed impairments in face processing in various psychiatric diseases such as major depression (Elliott et al., 2011), schizophrenia (Kohler et al., 2010), or alcoholism (Maurage et al., 2008). However, studies on auditory deficits are much less frequent, and multisensory emotion processing studies in clinical populations are largely missing, even though recent findings indicate that impairments in emotion integration may be equally important. This is nicely illustrated for the example of alcoholism in the review of Maurage and Campanella (2013). Complex emotional designs can also identify neural similarities between disorders. Using an audiovisual emotion paradigm, Müller et al. (2013) showed that both schizophrenia and depressive patients had a dysfunctional regulation in the same region of the angular gyrus. Even for subclinical deficits in emotion perception skills, multimodality may be the crucial factor. Delle-Vigne et al. (2014) investigated the processing of complex audiovisual stimuli in relation to alexithymia scores. Specifically for bimodal emotions, high alexithymic participants had higher amplitudes in the P100 and N100 components. This could not be observed in studies using unimodal stimulation.

A study by Zvyagintsev et al. (2013) addressed an aspect of integration which is particularly relevant for schizophrenia patients: the suppression of task-irrelevant information. Patient ratings of visual stimuli were influenced by concurrent auditory information. This was the case for emotional and non-emotional material, indicating that modality-specific selective attention is disturbed in schizophrenia already at early sensory levels. Interestingly, healthy controls showed a similar effect solely for emotions, demonstrating an attentional capture effect across modalities. This is supported by the study of Adolph et al. (2013), showing that chemosensation interacts with visual perception. Here, the perception of sweat enhanced the allocation of attention to anxious faces. Moreover, sweat from social anxiety situations enhanced the processing of fearful facial stimuli only in socially anxious individuals—an impressive example of the integration of fear-relevant cues being influenced by personality traits. The interaction of visual emotion processing with irrelevant auditory cues was also subject of the study by Wolf et al. (2014). The authors demonstrated that visual emotion cues modulated tone processing in the auditory cortex. Thus, affective information in one sensory domain can influence even primary sensory cortex areas of another modality. Although there is overwhelming evidence for a functional specialization of sensory cortices, this contributes to the growing body of investigations suggesting that there is no cortex area which can be influenced solely by one sensory channel. Emotional content thus can trigger this crossmodal modulation. In a similar way, auditory emotional cues can enhance early cortical processing of visual stimuli. Gerdes et al. (2013) found an amplitude modulation of early visual P100 and P200 components when pictures were accompanied by emotional sounds. Emotional “crosstalk” between early auditory and visual areas thus seems to exist in both directions.

Emotional content can also modulate multisensory integration areas. Whereas matching affective information in different channels facilitates emotion recognition, non-matching information leads to emotional conflict. Watson et al. (2013) showed that audiovisual integration areas of the superior temporal cortex are sensitive to emotional congruency: Conflicting affective information enhanced activity in these sensory integration areas. Stronger cortical processing of incongruent emotional stimuli was also reported by Gerdes et al. (2013). They found enlarged P100 and P200 components for conflicting emotional information. Emotional sounds thus seem to modulate visual processing as early as 100 ms after stimulus presentation. These early interactions may be due not only to sensory integration, but also to crossmodal prediction. In real life, affective information from e.g., face and voice often do not arrive in perfect synchrony at the recipient's eyes and ears; one modality often precedes the other one. Information from the earlier modality forms an expectation about the emotion in the other sense and modulates processing accordingly in a top-down fashion. Jessen and Kotz (2013) comprehensively review the literature on emotional crossmodal prediction and highlight its importance for stimulus integration.

Recent studies identify the amygdala and adjacent anterior temporal lobe structures as central for emotion evaluation and integration (Klasen et al., 2011; Mathiak et al., 2011). This is also highlighted in a lesion patients study by Milesi et al. (2014). Their findings confirm the role of the amygdala and anterior temporal lobe as parts of the visual system, but also show their importance for evaluating particularly positive emotional stimuli across modalities. Moreover, these data show that a lacking ability to identify emotions in one domain can be compensated by cues from another. The same seems to be true in healthy controls when emotional information in one channel is missing. Regenbogen et al. (2013) investigated neural responses in various brain areas during video clips with emotional information in face, prosody, and speech content. If emotion from one channel was missing, input from the dorsomedial prefrontal cortex to the respective sensory cortex areas was increased, indicating a top-down modulation filling the sensory gap. The role of the amygdala for emotion processing was also highlighted in a multimodal fear conditioning study by Sripada et al. (2013). They investigated fear extinction processes in war veterans suffering from PTSD. Hyperactivation in fear-related brain circuits encompassing the amygdala during fear extinction was related to avoidance symptoms.

An important contribution to basic research with clinical perspectives is delivered by Kreifelts et al. (2013). They investigated the impact of emotion communication training on brain structure and function. Emotion-specific training modulated activity in cortical areas of face and voice processing, which shows their importance for emotion evaluation. Structural changes, however, were observed only in the fusiform face area (FFA). These findings support the notion that visual and auditory modalities support each other when emotions are categorized, but they also highlight the dominant role of vision. Visual dominance in emotion processing was also reported by Regenbogen et al. (2013). Here, the presence of facial emotions enhanced functional connectivity between the FFA and areas of the angular gyrus associated with audiovisual speech integration (Bernstein et al., 2008). Neural systems thus seem to prioritize emotional over neutral facial information. However, no such effect was observed for vocal emotions or auditory cortex. In a similar vein, Sestito et al. (2013) reported a prioritization of visual over auditory information for incongruent face-voice pairings. This was also reflected in autonomously triggered facial mimicry: Visual emotions led to stronger facial reactions than auditory ones. Peripheral physiological reactions triggered by affective signals play a decisive role in the genesis of emotional states (Brouwer et al., 2013). Accordingly, facial muscle reactions to emotional cues are reduced in schizophrenia patients who show emotion recognition impairments (Sestito et al., 2013). Taken together, visual information is more important than auditory for judging emotions; accordingly, bimodal emotional stimuli are primarily classified by their visual content (see also Klasen et al., 2011). This consistently reported prominence may in part be attributed to an unspecific visual dominance effect (Colavita, 1974); however, in the case of emotional cues, the fact that auditory signals are less reliable than visual ones may also add to the picture (Klasen et al., 2012a).

Recent evidence shows that interactions between emotional information are not limited to hearing and vision. Frank et al. (2013) discuss the multisensory integration of food-related cues in the insular cortex. Being a multimodal cortex region, the insula has been described as integrating interoceptive states with contextual information (Craig, 2009). Deviant stimulus processing in the insula has been discussed as the neural basis of various eating disorders; Frank et al. (2013) discuss the clinical implications of this association. Being essential for the processing of food-related stimuli, the insula has been related to the processing of disgust-related stimuli from various modalities (Jabbi et al., 2008). The evolutionary significance of this function is obvious; checking if something is edible or spoiled relies on smell, taste, vision, and touch. The insula supports the integration of this information and thus seems to contribute essentially to the feeling of disgust. Accordingly, Croy et al. (2013) showed that disgust could be evoked via visual, auditory, tactile, and olfactory stimulation. Peripheral responses such as blood pressure, heart rate, or galvanic skin response, however, varied with modality.

An extraordinary, but important aspect of multisensory integration is investigated by Bensafi et al. (2013) and Ohla and Lundström (2013): the interaction between olfactory and trigeminal stimuli. These modalities are closely intertwined; in real life, there is almost no smell which does not trigger both systems. This sensory interplay is of high relevance for our perception of food and drinks. Bensafi et al. (2013) found shorter latencies of N1 and P2 responses and reduced N1 amplitudes to combined olfactory and trigeminal stimuli compared to both modalities in isolation. These findings suggest that trigeminal and olfactory cues support each other and reduce neural processing workload—in analogy to the findings from other modalities. Moreover, the authors identified the rostral anterior cingulate cortex as a binding region for olfactory and trigeminal stimuli. In a second study, Ohla and Lundström (2013) investigated gender effects in olfactory-trigeminal integration. The authors demonstrated that, despite comparable sensory sensitivity, women perceived trigeminal stimulation as more irritating than men. This was also reflected in enlarged late positive EEG components. These findings show a differential integration of olfactory and trigeminal stimulus aspects in men and women.

Recognizing that emotional experience in real life is a multisensory phenomenon leads to the conclusion that approaches using unimodal or static stimuli often lack external validity. This problem has been addressed by complex stimuli and innovative experimental designs. Another novel approach was applied by Wilson-Mendenhall et al. (2013). They employed the multisensory imagination of scenarios leading to negative emotion experience. This procedure creates actual emotional experience based on situational information and goes beyond reactive stimulus processing. Moreover, it takes into account that real-life emotional experience is not limited to some basic emotions and often goes far beyond a one-way stimulus-response pattern. Since the core function of emotions is guiding the individual's behavior via motivational processes, humans tend to actively search situations evoking positive emotions and to avoid situations associated with negative emotional outcomes. These degrees of freedom are difficult to realize in a traditional experiment. Virtual reality settings provide a promising tool for studying affective processes in multimodal environments. They are close to reality and allow the participants to individually select their actions based on rewarding values. Recent fMRI investigations have shown that video game paradigms are well suited to study the brain correlates of realistic behavior patterns using fMRI (e.g., Mathiak and Weber, 2006; Mathiak et al., 2011; Klasen et al., 2012b, 2013). Kätsyri et al. (2013) investigated responses of the brain reward system to different types of events during free play of a multimodal violent video game using fMRI. They found that win and loss events differentially affected midbrain structures of the mesolimbic reward system; however, these effects did not predict subjective measures of emotional experience. Such insights into the neural processes underlying situational experience in video games come from the study by Mathiak et al. (2013). The authors used a combined approach integrating both game content and measures of game-induced affect. Their findings highlight the importance of cortex areas involved in self-referential emotion processing for the experience of more complex emotions in the virtual environment. Taken together, these findings indicate that reward-motivated behavior is strongly determined by striatal activity; the cognitive appraisal component which leads to perceived emotions, however, relies on cortex areas dedicated to the representation of inner states.

In summary, the investigations presented in this volume show that emotions from different senses interact at multiple levels, influence each other, and form holistic percepts, involving a variety of brain structures from unisensory cortices to high-level association areas. Importantly, they also clearly point out that emotional perception involves all human senses—not only hearing and seeing, but also touch, smell, taste, and even trigeminal signals. Moreover, they highlight the crucial necessity of taking into account the factor of multimodality when the neural processing of emotional situations is investigated.

## Conflict of interest statement

The authors declare that the research was conducted in the absence of any commercial or financial relationships that could be construed as a potential conflict of interest.
